# How Neurologists Combine Clinical Signs and Subjective Factors to Diagnose Epileptic and Functional Seizures: Insights From Seizure Video Analysis

**DOI:** 10.1002/brb3.70866

**Published:** 2025-09-16

**Authors:** Subramanian Muthusamy, Udaya Seneviratne, Henry Ma, Thanh G. Phan

**Affiliations:** ^1^ School of Clinical Sciences at Monash Health, Department of Medicine Monash University Melbourne Victoria Australia; ^2^ Department of Neurology Monash Medical Centre Clayton, Melbourne Australia

**Keywords:** diagnosis, epileptic seizure, functional seizure

## Abstract

**Background:**

Evaluating seizure semiology is crucial for distinguishing functional seizures (FS) from epileptic seizures (ES). This study examines how neurologists integrate clinical signs, observations, and subjective factors in making this distinction.

**Methods:**

Neurologists at Monash Health assessed unlabeled seizure videos on a web‐based platform, providing free‐text descriptions, selecting observed clinical signs, offering diagnoses, and rating their certainty (0–100). Non‐negative matrix factorization (NMF) clustered clinical signs, and thematic analysis was performed on free‐text responses. Logistic regression evaluated how single signs, sign combinations, and rater consensus influenced diagnostic alignment. Model performance was assessed using area under the receiver operating characteristic curve (AUC).

**Results:**

NMF identified two sign clusters: one associated with FS (eye closure, asynchronous limb jerking, headshaking, pelvic thrusting) and another with ES (eyes open, head version, tonic/dystonic posturing, automatisms, synchronous limb jerking). Diagnostic alignment was influenced by clinical sign combinations (OR = 1.37, 95% CI = 1.15–1.64, *p* < 0.001) and rater consensus (OR = 1.40, 95% CI = 1.15–1.77, *p* = 0.002). Models incorporating these factors incrementally improved performance, with the final model achieving an AUC of 0.88 (95% CI: 0.84–0.92, McFadden's *R* ^2^ = 0.37).

**Conclusion:**

Neurologists rely on distinct clinical sign patterns and subjective factors to differentiate FS from ES. Understanding these decision‐making processes can improve diagnostic workflows and training programs.

## Graphical abstract

This study provides new insights into how neurologists integrate patterns of clinical signs and subjective factors (such as self‐rated certainty) to differentiate epileptic and functional seizures. Understanding these decision‐making processes can improve diagnostic workflows and training programs.

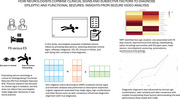

## 1 Introduction

Evaluation of seizure semiology—the sequence and pattern of clinical signs observed before, during, and after a seizure—is essential for differentiating functional seizures (FS) from epileptic seizures (ES) (Muthusamy et al. 2022). Accurate differentiation is critical for guiding treatment strategies and improving patient outcomes. Misdiagnoses can result in inappropriate antiepileptic drug treatments for patients with FS or delayed psychological and behavioral interventions (Asadi‐Pooya and Tinker 2017; Doss and LaFrance 2016; Russell et al. 2016). This diagnostic process requires neurologists not only to identify individual clinical signs (e.g., eye closure, head version) but also to integrate these signs within the broader sequence of clinical signs that occur during the seizure.

While individual clinical signs such as eye closure or asynchronous limb jerking have been previously associated with FS, these signs have typically been studied in isolation (Bounds 2007; Brigo et al. 2013; Chen et al. 2008; Chung et al. 2006; DeToledo and Ramsay 1996). This approach does not reflect how neurologists make diagnostic decisions in real‐world practice, where they must synthesize multiple, often co‐occurring, features. Moreover, subjective elements such as certainty in one's diagnosis may also influence diagnostic accuracy but have received limited attention in seizure semiology research. This study explores how neurologists integrate clinical signs and subjective factors to differentiate FS from ES. We hypothesize that (1) combinations of clinical signs, rather than individual signs, will be stronger predictors of diagnostic accuracy and (2) the complementary integration of multiple factors (e.g., self‐assessed certainty) will further enhance overall diagnostic performance.

## 2 Methods

Videos of 11 ES from 11 patients and 11 FS from 11 patients were selected from video EEG monitoring (VEM) recordings. The survey was designed with careful consideration of the participating neurologists' time constraints. The number of videos included was similar to a previous study (Seneviratne et al. 2012). Seizure videos incorporating a mixture of ES and FS semiology from consenting patients were selected based on (1) visible semiology and (2) video quality sufficient to discern clinical manifestations. Written consent was obtained from all patients. These 22 patients were diagnosed with ES or FS based on clinical assessment and VEM data by epileptologists’ consensus. Seizures were classified according the International League Against Epilepsy (ILAE) classification of seizure types for ES and classification system proposed for FS according to this study (Fisher et al. 2017; Seneviratne et al. 2010).

Study data were collected and managed using REDCap electronic data capture tool hosted and managed by Helix (Monash University), a data management platform (Harris et al. 2019; Harris et al. 2009). Each video, approximately 30–45 s long and unaccompanied by clinical or EEG data, was followed by questions that required participants to (1) describe the clinical event, (2) select (tick) observed clinical signs (e.g., eye closure, pelvic thrusting), (3) provide the most likely diagnosis (e.g., ES, FS, other, unsure), and (4) self‐assess certainty in the diagnosis as a score (ranging from 0 to 100). A cropped video showing observable seizure manifestations was used instead of the full seizure video due to the size limitations for uploads onto the REDCap platform. See Appendix 1 for a detailed list of the questions. All participants were neurologists from our institution (due to institutional ethics constraints) who were not involved in the care of patients whose videos were included. There was no time restriction on completing the questions, and participants could complete it in multiple sittings, although completion in one sitting was encouraged. Participants could review each video as many times as needed, but once they submitted responses to questions related to a particular video, responses could not be altered.

### 2.1 Statistical Analysis

Statistical analyses were performed using R statistical programming language (version 4.1.0). Descriptive statistics were used to summarize rater characteristics (e.g., years of experience), patient characteristics (e.g., age, seizure semiology, and VEM diagnosis), rater diagnoses, and self‐assessed rater certainty scores. Rater experience was categorized into three groups (0–5 years, 5–10 years, and more than 10 years), while self‐assessed rater certainty scores were grouped into three categories (0%–50%, 50%–75%, and 75%–100%). The number of observed clinical signs was summarized for each diagnosis and visualized using boxplots.

Logistic regression analysis was conducted to model the likelihood of a correct diagnosis based on key predictors, including single sign versus the same single sign but in combination with other signs, rater consensus (number of raters agreeing on a combination of observed clinical signs for each case), and self‐assessed rater certainty (certainty category: 0%–50%, 50%–75%, 75%–100%). We calculated area under the receiver operating characteristic curve (AUC) for baseline models (single clinical sign) across all clinical signs, allowing for the identification of the clinical sign with the highest discriminatory ability. Models were then constructed incrementally, beginning with the baseline model and sequentially adding additional covariates: clinical sign combinations involving the chosen clinical sign observed at least five times (to ensure clinical relevance), rater consensus, and self‐assessed certainty category. The differences in AUC between successive models were calculated using *z*‐scores. To address multiple comparisons, *p* values were adjusted using the false discovery rate (FDR) correction (Glickman et al. 2014). McFadden's pseudo *R* ^2^ was calculated for each model to assess model fit (Hemmert et al. 2018), with values ranging from 0 to 1. Higher pseudo *R* ^2^ values indicate better fit, with thresholds suggesting weak (0.02–0.06), moderate (0.07–0.20), and strong (above 0.2) model performance.

### 2.2 Non‐Negative Matrix Factorization

Non‐negative matrix factorization (NMF) is an unsupervised clustering method that has been successfully used in a wide range of applications including text mining, image analysis, and phenotyping (Guo et al. 2024; Lee and Seung 1999). We utilized NMF as follows.

### 2.3 Finding Patterns of Clinical Signs

Raters’ assessments of clinical sign presence or absence were compiled into a data matrix representing average responses (Campagner et al. 2021). The optimal number of clusters was determined using several metrics, including cophenetic coefficients (measures how consistently data points cluster across iterations), dispersion and silhouette width (reflect the compactness and separation of clusters), and reconstruction error (which indicates the accuracy of data reconstruction) (Hutchins et al. 2008, Brunet et al. 2004). The results of NMF were visualized using three types of heatmaps:

1. Basis heatmaps: These show which clinical signs belong to which cluster (columns), highlighting the degree of contribution (“weight”) each sign makes to a given cluster. This informs which features are most representative of each group.
2. Coefficient heatmaps: These illustrate how strongly each patient's seizure presentation corresponds to the identified clusters (rows). Each column represents a patient, and the relative weights across clusters indicate which semiological pattern best describes that case.
3. Consensus heatmaps: These visualize the stability and reproducibility of clustering assignments across multiple NMF runs. Higher consensus values between sign‐pairs suggest those signs frequently cluster together, confirming the robustness of the groupings.

Feature importance scores were extracted from the basis matrix to identify the most influential signs in each cluster. Subgroup analyses compared cluster patterns across neurologists grouped by clinical experience.

### 2.4 Thematic Analysis of Free‐Text Responses

NMF was applied to the free‐text responses of raters to cluster clinical sign terms. This approach analyzes patterns of word co‐occurrence across raters’ descriptions, grouping related terms into clusters, allowing for the discovery of hidden thematic structures in the free‐text data. Since averaging responses was not feasible, all individual responses were included in the analysis. The results were visualized as horizontal bar graphs, showing the 10 most frequent terms within each cluster (or topic) alongside their importance values (extracted from the basis matrix). Subgroup analysis was performed to compare clusters across experience groups.

### 2.5 Institutional Ethics Approval

Institutional ethics approval was obtained for this study (NMA HREC Reference Number: LNR/18/MonH/181). Only Monash Health neurologists could participate in the study.

## 3 Results

A total of 22 neurologists (out of 33 invited) completed the survey. None were epileptologists. The median number of years of experience as a neurologist was 12.5 (range: 1–33), with five neurologists having 0–5 years of experience, five having 5–10 years, and 12 having more than 10 years of experience. Patients included in the study had a median age of 33 years (range: 18–76), with 55% being female (60% in the FS cohort and 50% in the ES cohort). The details of seizure semiology, classification, and diagnosis for ES are provided in Table 1.

**TABLE 1:** Summary of seizure description, classification, diagnosis and seizure focus.

ID	VEM description	Seizure classification	Diagnosis	Focus
1	In this episode, there was rhythmic rocking of the lower limbs associated with mouth puckering and cooing noise.	Complex motor FS	FS	NA
2	The patient is awake lying in bed. He touches his face with the left hand. Then, there is tonic head version to the right side followed by generalized stiffening of arms and legs. He goes into fencing posture with extension of right upper limb and lower limb and flexion of left upper limb at elbow and left lower limb at knee and hip. This is followed by flexion of right leg and elevation of left upper limb. Clonic movements are seen in both upper limbs and lower limbs. This is followed by figure of 4 sign with flexion at the right elbow and straightening of the left upper limb. The patient goes into generalized tonic‐clonic seizure. Last clonic jerks are seen involving the left upper limb.	FBTCS	Focal epilepsy	Left temporal
3	Patient is awake, sitting up in bed, watching TV. The clinical onset is characterized by a sudden body jolt followed by flapping‐type movements of the right arm and dystonic posturing of the right upper limb. Then, there is tonic head version to the left followed by generalized stiffening and bilateral convulsive movements before she becomes postictal.	FBTCS	Focal epilepsy	Left temporal
4	The first semiological sign visible at seizure onset is left upper limb movement. Usually, there are three to four repetitive movements at the shoulder and elbow joints in the form of flexion‐extension and rotation. In some seizures, dystonic posturing of the right upper limb is visible. Oral automatisms characterized by repetitive chewing are seen. This is accompanied by bilateral flexion‐extension hip movements, knee flexion, and pelvic thrusting. When all movements settle down, there are fast flapping‐type movements of right hand as the last semiological sign before seizure termination.	Focal motor	Focal epilepsy	Left temporal
5	The events start with her eyes closed. She rolls her head from side to side then starts hyperventilating. The breathing becomes faster gradually. Then she starts back arching. This is followed by cycling type of movements in both lower limbs with intermittent pelvic thrusting. The cycling movements settle down and irregular arrhythmic jerking of the legs can be seen. The event comes to an abrupt end.	Hypermotor FS	FS	NA
6	Patient is lying in bed reading a book. She puts the book down and right leg starts jerking slowly. Then, it becomes bilateral, and jerking is irregular, asynchronous, and asymmetrical. One minute later, there is left hand flapping followed by rhythmic pelvic thrusting. Gradually, bilateral leg jerking becomes faster. The nurse enters the room and removes spectacles. The patient's eyes are closed, and she is unresponsive. Gradually, she starts responding to the nurse.	Complex motor FS	FS	NA
7	Heavy breathing, irregular body jerking in fetal position with pelvic thrusting. Eyes open. Arose from wakefulness.	Complex motor FS	FS	NA
8	Tonic posturing including head, elbow, and knee flexion. During the episode, he lay on his back with his arms flexed and fingers adducted. The head deviated to the right. (Another episode described as: comprised tonic posturing, with flexion of both legs and arms.)	Generalized tonic	EE (generalized seizure)	NA
9	These events are characterized by irregular asymmetric, asynchronous, and arrhythmic jerking of arms and legs. Sometimes, this is associated with facial grimacing, and axial jerking as well. Sometimes, there are twisting contortions of the arms and legs. Each event is very brief, lasting only a few seconds.	Complex motor FS	FS	NA
10	The patient is lying in bed awake. He presses the seizure button. Then his right leg starts shaking very fast. His eyes are closed. Right leg shaking is followed by whole body trembling. The arm and leg jerking are asymmetric and asynchronous. The frequency and amplitude vary from time to time. The shaking is irregular. Occasional hip flexion is also seen.	Mixed (rhythmic + complex) FS	FS	NA
11	Awake but eyes closed at start of seizure. Patient opens eyes and looks at his left hand. There is hypermotor activity of both feet. He rubs his hands, then covers his face with both hands. There is right finger pointing, and he says something, but the audio is not clear. Hypermotor activity of both lower limbs is once again noted. Next, we see ictal pouting. He subsequently climbs over the edge of the bed and pulls at the electrodes. He walks over to the other side of the bed outside the view of the camera. He pulls off the electrodes and places leads on the bedside table. He picks up facial tissues to wipe his head and face. He picks up electrodes and walks to the other side of the room and gets onto the bed by climbing the siderails. He is then noted to be looking confusedly at electrodes as well as intravenous cannula.	Hypermotor ES	Focal epilepsy	Left frontotemporal
12	She is collapsed to the left side and remains unresponsive. She demonstrates violent jerking similar to previous clinical events. She is unresponsive during the episode, and her eyes are closed.	Hypermotor FS	FS	NA
13	Patient is lying on right side with her knees flexed. There is irregular jerking movements of all her limbs and the left leg, and the left foot is held over the bed rail.	Complex motor FS	FS	NA
14	At the onset, eyes are closed. The episode commencing with right hand tapping followed by onset of left leg shaking and pelvic thrusting. The amplitude of the movements increased and evolved to include both legs and continued asynchronously. The head turned toward the right side. There was also development of non‐rhythmic head shaking and both arm shaking. These movements eventually slow down and cease. The eyes remain closed throughout the episode. Following the offset of symptoms, patient was unresponsive to voice and painful stimuli. Later, patient's eyes open spontaneously and turned onto her right side. Later, the patient awakens and is able to verbalize and proceeds to get out of bed.	Hypermotor FS	FS	NA
15	Lying in the right lateral position, awake and looking at mobile phone, tonic extension of the neck and frog‐like extension of the limbs, this is followed by loud moaning and complete extension of all limbs and generalized clonic movements and eye lid flickering. This event lasted 1 min, and there was considerable pallor noted and subsequent hypoxic tonic and myoclonic activity.	GTCS	IGE	NA
16	The patient is lying in bed. The left foot starts twitching which stops after a few seconds. Subsequently, the left arm starts jerking. This is followed by left leg jerking intermittently. This is associated with side‐to‐side head shaking. His eyes are closed. For about a minute, intermittent right leg jerking can be seen. The amplitude and frequency of these movements vary from time to time. The patient remains unresponsive during the episode when the nurse talks to him. Later, the movements stop, and he responds.	Complex motor FS	FS	NA
17	Characterized by vigorous head nodding and body shaking. The shaking of the arms and legs are asymmetric, asynchronous, and irregular. The frequency is variable, and it becomes very fast toward the end of the seizure. Occasionally, side‐to‐side head shaking is also seen. The patient is unresponsive during the episode. Toward the end of the seizure, pelvic thrusting and violent flaying movements are seen. This seizure arises from the awake state. Other events characterized by irregular jerking of arms, legs and the trunk associated with fast pelvic thursting. Head nodding is also seen.	Complex motor FS	FS	NA
18	The patient is watching his computer on his back. The arms are flexed over his chest. Oral automatisms are seen.	Focal motor automatisms	Focal epilepsy	Left temporal
19	Eyes are open, the head is extended, and the whole body stiffens and jerks, with right arm extension.	Focal motor tonic	Focal epilepsy	Left temporal
20	Rolls over in bed, smacked her lips.	Focal motor automatisms	Focal epilepsy	Right temporal
21	Strokes her face and forehead and the back of her hair with her right hand, the left arm is mostly fixed behind the head, but as the seizure progresses, both arms are resting on her abdomen. She appears unresponsive. Toward the end of the seizure, there were slow jerky movements of both upper limbs with oral automatisms, the left arm was flexed and there was prominent left facial twitching and jerking of the left arm and fingers. The focal left facial twitching became more prominent with head deviation to the left before secondary generalization with clonic activity.	FBTCS	Focal epilepsy	Right posterior quadrant
22	His eyes open, there is a change in facial expression, he seems to grimace, he opens his eyes, wipes the right side of his nose and the hands return clasped together. Head slowly turns to the right and the right arm flexes. His body quickly turns to the left, before returning to the bed, fully extended, with his head still deviated to the right, his elbows flexed and his hands clenched. Rhythmic jerking begins the left leg, then the torso and the right leg, his face is very red, his head remains extended back against the bed, clonic movements begin in the left hand and right arm, and the hands gradually then drop towards the side of this abdomen. Facial grimacing continues. His hands are now rhythmically touching the sheets, before he becomes relatively motionless and the event terminates.	FBTCS	Focal epilepsy	Left temporal

Abbreviations: EE, epileptic encephalopathy; FS, functional seizures, FBTCS, focal to bilateral tonic clonic seizure, VEM, video EEG monitoring.

### 3.1 Self‐Assessed Rater Certainty in Diagnosis

This varied by case and seizure subtype. Self‐assessed certainty was the highest for tonic‐clonic seizures (97%) and the lowest for focal motor seizures with automatisms (68%) or hypermotor ES (71%) (Tables S1 and S2). By experience level, median self‐assessed certainty was 68% for neurologists with 0–5 years of experience, 80% for those with 5–10 years of experience, and 74% for those with more than 10 years of experience. Alignment with the true diagnosis was also assessed by experience level. Raters with 0–5 years of experience aligned with the true diagnosis in 75% of cases, those with 5–10 years in 83%, and those with more than 10 years in 77% (*p* = 0.382).

### 3.2 Tick Box Counts by Diagnosis

**FIGURE 1:**
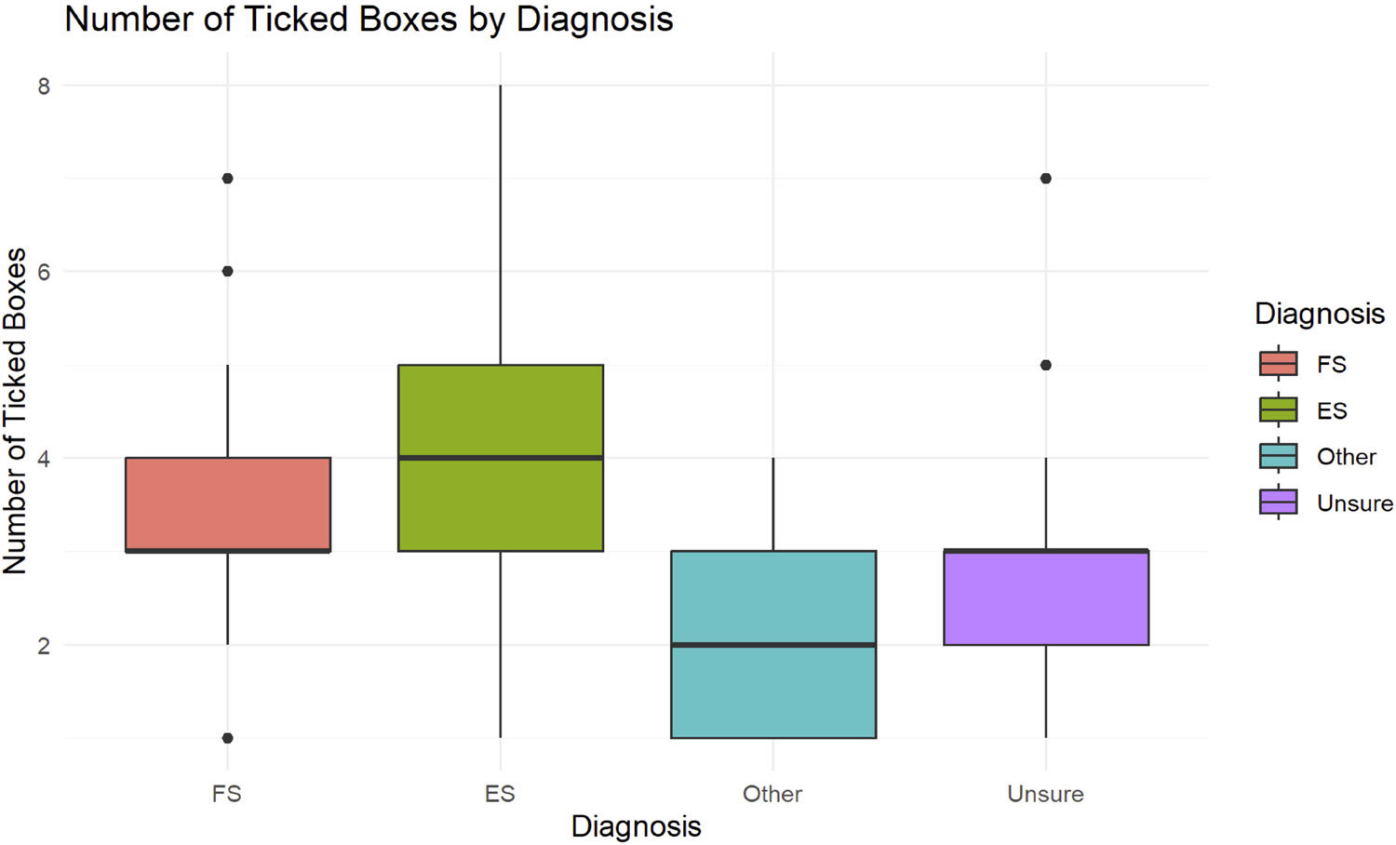
Distribution of ticked clinical signs by diagnosis. Boxplot shows the number of ticked clinical signs (structured annotations) across the diagnostic categories: epileptic seizures (ES), functional seizures (FS), other diagnoses, and unsure. On average, neurologists selected more clinical signs for ES and FS cases compared to “Other” or “Unsure” diagnoses, with the narrowest distribution observed in “Unsure” cases.

The number of observed (ticked) clinical signs differed significantly by diagnosis. Raters selected more clinical signs on average for cases diagnosed as ES (mean: 7, standard deviation [SD]: 2) and FS (mean: 5, SD: 1.8) compared to cases categorized as “Other” or “Unsure,” with “Unsure” diagnoses showing the narrowest distribution (Figure 1).

### 3.3 Predictors of Diagnostic Accuracy

Significant predictors of diagnostic accuracy included multiple sign combinations (odds ratio [OR] = 1.37, 95% confidence interval [CI] = 1.15–1.64, *p* < 0.001), rater consensus (OR = 1.40, 95% CI = 1.15–1.77, *p* = 0.002), and high self‐assessed rater certainty (75%–100%) (OR = 64.49, 95% CI = 30.21–149.25, *p* < 0.001). Among individual clinical signs, hyperventilation demonstrated the lowest discriminatory performance (AUC = 0.50, 95% CI = 0.48–0.52), while synchronous limb jerking and continuous temporal course achieved the highest AUCs (both AUC = 0.56, 95% CI = 0.51–0.60). After evaluating models incorporating all covariates across all clinical signs, eye closure, eyes open, and asynchronous limb jerking demonstrated the best model fit, achieving a McFadden's pseudo *R* ^2^ of 0.37.

The baseline model using eye closure achieved an AUC of 0.53 (95% CI: 0.48–0.58), reflecting limited discriminatory performance, with a pseudo *R* ^2^ of 0.002. After evaluating AUC across all models for all clinical signs, eye closure was selected as the initial predictor. Model 2, which included combinations of clinical signs involving eye closure, significantly improved diagnostic accuracy (AUC = 0.69, 95% CI: 0.63–0.75, pseudo *R* ^2^ = 0.07, *Z* = −4.27, *p* < 0.001). Clinical sign combinations in Model 2 were restricted to those observed at least five times, ensuring the inclusion of clinically relevant and commonly observed patterns. Model 3 incorporated rater consensus, further enhancing performance with an AUC of 0.72 (95% CI: 0.66–0.78, pseudo *R* ^2^ = 0.10, *Z* = −2.07, *p* = 0.05). The final model incorporated self‐assessed rater certainty (to previous co‐variates) and resulted in a substantial and statistically significant increase in AUC to 0.88 (95% CI: 0.84–0.92, pseudo *R* ^2^ = 0.37, *Z* = −5.88, *p* < 0.001). These findings are summarized in Table 2.

**TABLE 2:** Summary of the area under the curve (AUC) values, 95% confidence intervals (CIs), McFadden's pseudo *R* ^2^, *z*‐scores, and *p* values for logistic regression models assessing diagnostic accuracy using eye closure as the initial predictor.

Model	Covariates	AUC	95% CI	McFadden's pseudo *R* ^2^	*z*‐Score	*p* value
Baseline model	Eye closure	0.53	0.48‐0.58	0.002	NA	NA
Model 2	Eye closure clinical sign combinations	0.69	0.63–0.75	0.07	−4.27	<0.001
Model 3	Model 2 + rater consensus	0.72	0.66–0.78	0.10	−2.07	0.05
Model 4 (final model)	Model 3 + self‐assessed certainty	0.88	0.84–0.92	0.37	−5.88	<0.001

*Note: z*‐Score and *p* value were compared to the previous model. *p* values have been adjusted for multiple comparisons using the FDR correction. Eye closure was selected as the initial clinical sign after evaluating all five models across all clinical signs. The baseline model, which included eye closure alone, demonstrated limited discriminatory performance (AUC = 0.53, pseudo *R* ^2^ = 0.002). Model 2 evaluated combinations of clinical signs that included eye closure, resulting in a significant improvement (AUC = 0.69, pseudo *R* ^2^ = 0.07, *Z* = −4.27, *p* < 0.001). To ensure clinical relevance, combinations in Model 2 were restricted to those observed at least five times. Model 3 added rater consensus, further improving performance (AUC = 0.72, pseudo *R* ^2^ = 0.10, *Z* = −2.07, *p* = 0.05). The final model incorporated self‐assessed rater certainty and showed significant improvement (AUC = 0.88, pseudo *R* ^2^ = 0.37, *Z* = −5.88, *p* < 0.001).

### 3.4 Clinical Signs Clusters

**FIGURE 2:**
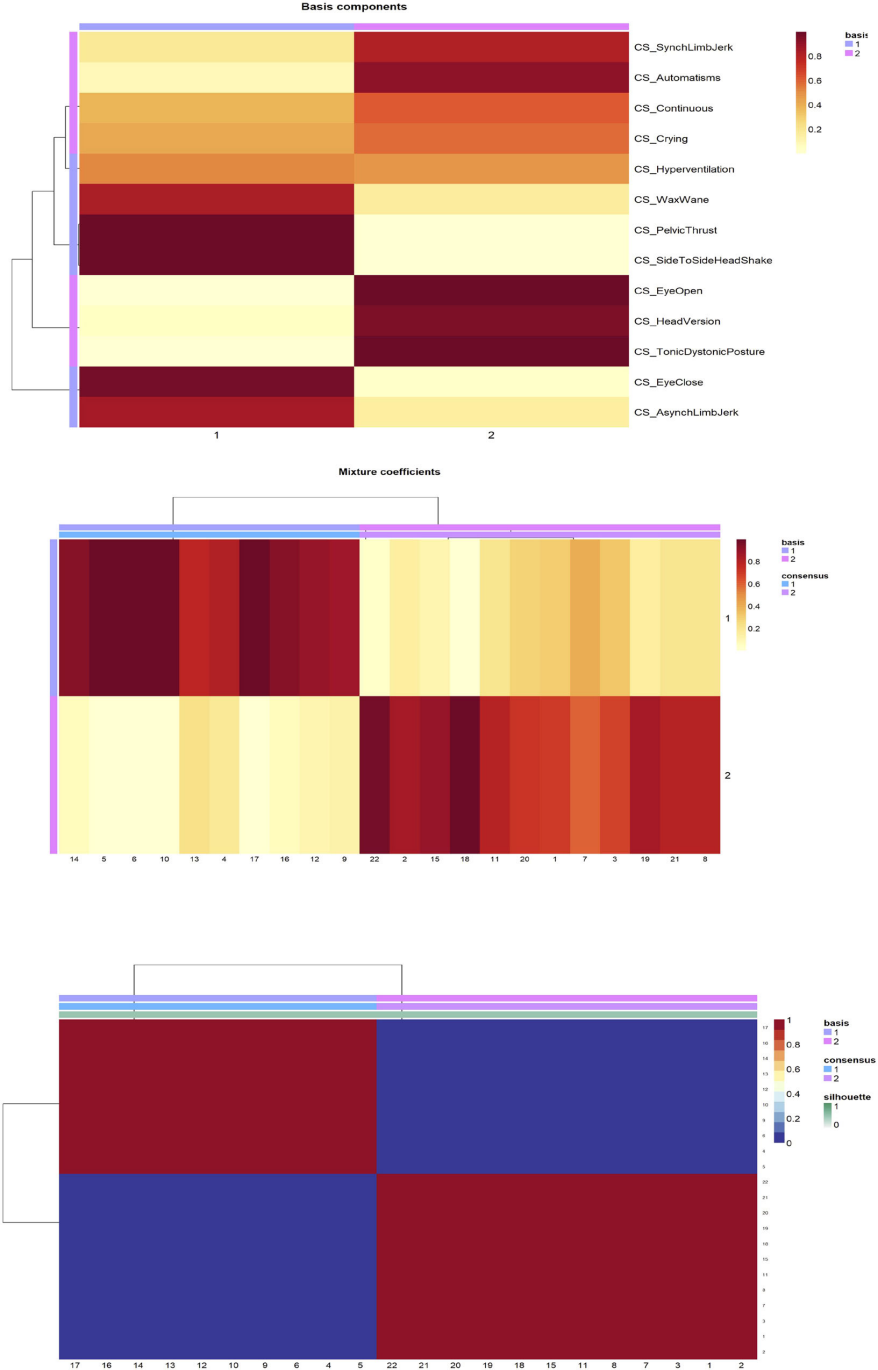
Clustering of clinical signs into two groups based on average rater response to the presence or absence of specific clinical signs. The “basis heatmap” represents the clustering of clinical signs into groups. The “coefficient heatmap” represents the “weights” attributed to each patient. The “consensus heatmap” visualizes the stability and consistency of clustering of the results across multiple NMF runs.

After testing various NMF algorithms (e.g., “Lee,” “Brunet,” “nsNMF”) and k‐ranks (2–11), the “Lee” algorithm was selected to cluster clinical signs into two groups (*k* = 2) based on its optimal metrics: cophenetic coefficient (1.00), dispersion (1.00), reconstruction error (3.73), and silhouette width (0.23). Cluster 1 included signs such as eye closure, asynchronous limb jerking, side‐to‐side headshaking, pelvic thrusting, and waxing‐waning temporal course. Cluster 2 included tonic or dystonic posturing, head version, eyes open during seizure, automatisms, and synchronous limb jerking (Figure 2). Feature importance values revealed that eye closure during seizure (0.14), asynchronous limb jerking (0.13), and tonic or dystonic posturing (0.11) were the most influential features in Cluster 1. In Cluster 2, eyes open during seizure (0.09) and head version (0.09) were the most important features.

The basis heatmaps for different experience groups are shown in Figure S1. The Basis heatmaps demonstrate similar clusters: one cluster included signs such as head version, synchronous limb jerking, eyes open during seizure, and tonic or dystonic posturing, while the other cluster included signs such as asynchronous limb jerking, eye closure during seizure, side‐to‐side head shaking, and pelvic thrusting.

### 3.5 Thematic Analysis of Free‐Text Responses

Using the “Lee” algorithm, thematic analysis of free‐text responses identified two distinct topics (co‐phenetic coefficient: 1.00, dispersion: 1.00, reconstruction error: 1030.75, silhouette width: 0.13). Topic 1 included terms such as jerking, asynchronous, eye closure, and pelvic thrusting, while Topic 2 was dominated by terms such as tonic, clonic, flexion, and grimacing (Figure S2). These clusters mirrored the semiological patterns observed in tickbox combinations, with terms like tonic‐clonic and asynchronous jerking providing additional context for ES and FS differentiation, respectively. The results for experience subgroups are shown in Figure S3.

## 4 Discussion

This study provides new insights into how neurologists integrate patterns of clinical signs and subjective factors to differentiate FS from ES. Although prior research, including our own meta‐analysis, has demonstrated the diagnostic value of individual signs (e.g., ictal eye closure or asynchronous limb jerking), these studies typically assessed each sign in isolation. In contrast, our study demonstrates that neurologists do not interpret signs independently but instead assess them as part of a broader behavioral context. Using multivariate modeling, we show that diagnostic accuracy improves substantially when co‐occurring signs are considered together and improves further with the inclusion of subjective elements such as self‐assessed certainty. This emphasizes the value of pattern‐based clinical reasoning, which more closely aligns with real‐world diagnostic practice. Additionally, our use of NMF to empirically identify clinical sign clusters that differentiate ES and FS represents a novel application of unsupervised machine learning methods within seizure classification research.

Our approach, which combined structured clinical signs and qualitative observations, allowed us to capture both rapid pattern recognition and more deliberate analysis. Analysis of tick box combinations revealed distinct patterns predictive of specific diagnoses (e.g., *eye closure*, *asynchronous limb jerking* and *pelvic thrusting* for FS, and *eyes open* and *automatisms* for ES) supporting the hypothesis that combinations of clinical signs, rather than individual signs, are stronger predictors of diagnostic accuracy. In parallel, free‐text descriptions provided valuable context, reflecting neurologists’ observational focus and offering deeper insights into their reasoning. For instance, terms such as *fluctuating*, *variable*, and *purposeful* were more frequently used in descriptions of FS than ES. These findings highlight key components of the clinical reasoning process (Heneghan et al. 2009), particularly the interplay between type 1 reasoning (intuitive recognition of familiar semiological patterns, as reflected in tick box responses) and type 2 reasoning (deliberate articulation of details through free‐text descriptions to refine or confirm diagnostic hypotheses) (Sloman 1996; Wason and Evans 1974).

We employed NMF to further enhance the understanding of clinical sign combinations in ES and FS. NMF can decompose data into interpretable components (Lee and Seung 1999) and uncover underlying structures within the clinical data that are not immediately apparent using conventional methods. Signs such as *eye closure*, *asynchronous limb jerking*, and *side‐to‐side head shaking* were clustered into one group, providing insights into clinical sign combinations observed during FS. Similarly, signs such as *eyes open during seizure*, *head version*, *tonic or dystonic posturing*, and *automatisms* were clustered into another group, which aligned with features associated with ES. While prior studies have only focused on clinical signs in isolation (Erba et al. 2016; Gröppel et al. 2000; Lombardi et al. 2020; Muthusamy et al. 2022; Syed et al. 2011), our analysis highlights combinations of signs that neurologists in this cohort considered diagnostically relevant. In complex cases, where symptom presentation might overlap between seizure types, identifying clinically relevant sign clusters could prove invaluable.

We also examined how the integration of additional factors could enhance diagnostic accuracy. Incorporating self‐assessed diagnostic certainty and rater consensus significantly improved the performance of our predictive models, reflecting the additive value of these factors in clinical reasoning. Self‐assessed diagnostic certainty, while a significant predictor, warrants further scrutiny. The high odds ratio for the 75%–100% certainty category (OR = 64.49) highlights a strong association with diagnostic accuracy but also raises concerns about its reliability and potential overestimation. Although self‐assessed certainty is a key element of clinical decision‐making, it may sometimes reflect overconfidence rather than true diagnostic skill. Overconfidence can be amplified in cases with prototypical semiology (e.g., tonic‐clonic ES), where familiarity may reinforce subjective certainty (Cassam 2017). Conversely, in ambiguous or complex cases, high confidence may not correspond to diagnostic accuracy due to cognitive biases, such as confirmation bias or availability heuristics (Berner and Graber 2008). The strong association observed in this study may thus reflect two distinct scenarios: confidence justified by clear clinical patterns and over‐reliance on intuition in the absence of systematic analysis. Balancing subjective confidence with objective data (e.g., clinical sign clusters) may offer a more reliable framework for decision‐making.

The variation in diagnostic certainty across seizure types has important clinical implications. Focal seizures with automatisms were associated with lower rater confidence, likely due to their lack of overt or stereotyped features. In the absence of concurrent EEG or detailed clinical history, these presentations can be more challenging to recognize and accurately classify. In contrast, hypermotor ES often present with unusual semiology, which may be misinterpreted as FS, even by neurologists (Peter et al. 2024). These cases highlight the potential risk of underdiagnosing or misclassifying focal ES with subtle or complex phenomenology. Early referral to an epileptologist and timely access to video EEG monitoring should be prioritized in such cases to avoid diagnostic delays.

Although our raters answered questions about seizure videos independently, the finding that consensus is a significant predictor of diagnostic accuracy emphasizes its usefulness in clinical practice. In real‐world settings, diagnostic consensus often emerges through case discussions or multidisciplinary meetings, where clinicians share perspectives and interpretations of semiological patterns. Consensus‐based approaches have been shown to enhance diagnostic accuracy by leveraging collective expertise and diverse perspectives. These methods can mitigate individual biases and errors (Pilgrim et al. 2024; Tversky and Kahneman 1974), particularly in complex or uncertain cases where no single observer has complete information (Kane and Luz 2009). In the context of FS and ES differentiation, consensus may reduce variability in the interpretation of semiology, enabling more accurate diagnostic decisions.

Our findings have several potential implications for clinical practice and training. First, it affirms the need to teach clinicians not to rely on individual clinical signs in isolation but to interpret seizures based on combinations of co‐occurring signs. Second, while confidence in diagnostic decisions can reflect experience and familiarity, our data highlight the importance of remaining cautious about overconfidence, particularly in ambiguous cases. Third, it reinforces the value of collaborative diagnostic discussions, as rater consensus was associated with greater diagnostic accuracy. In situations where focal seizures have subtle or ambiguous features, or where diagnostic certainty is low, clinicians should consider early referral for video EEG monitoring or evaluation by an epileptologist to avoid misdiagnosis. While preliminary, these findings can inform future efforts to optimize training programs, enhance clinical reasoning, and improve diagnostic pathways for seizure classification.

### 4.1 Limitations

Our study has several limitations. First, the videos used in our survey were unaccompanied by clinical data. While this deviates from typical clinical practice, this was an intentional design decision to isolate the diagnostic contribution of semiology. We acknowledge that this limits direct extrapolation to clinical settings. However, our study allows a clearer understanding of how neurologists assess semiology as a standalone diagnostic tool. Second, the list of clinical signs presented to participants was predefined. While this standardization ensured consistency in responses and enabled quantitative analysis, it may have influenced raters' attention, potentially biasing them toward signs included in the list and away from unlisted features they might otherwise have considered. Third, none of the clinician raters were epileptologists. This was also by deliberate design. The epileptologists at our institution were involved in the clinical care or diagnosis of one or more patients and were therefore excluded to avoid potential bias. We also sought to understand the diagnostic reasoning of general neurologists, who may be more likely to assess seizure presentations in outpatient or emergency settings. To mitigate variability among raters, we standardized the survey using a structured checklist of clinical signs and used a composite data matrix to account for inter‐rater variability. Fourth, the relatively small sample size of 22 videos limits the generalizability of our findings. However, we wanted to ensure that the survey remained feasible for participating neurologists while maintaining engagement and data quality. Although a larger video set may have offered additional insights, we prioritized methodological rigor and completion rates. Fifth, videos were pre‐selected based on visible semiology and adequate video quality, which may introduce selection bias. This strategy, while necessary to enable reliable clinical sign assessment, may have favored more overt or stereotyped seizure presentations and limits applicability to more subtle, ambiguous, or poorly visualized cases. For example, we were unable to evaluate how neurologists interpret vocalizations or non‐observable features such as auras. Finally, all clinician participants were neurologists from a single institution, as constrained by ethics approval. While this may limit external validity, the study nonetheless offers a foundation for understanding diagnostic reasoning in seizure classification. Future research should involve larger, more diverse videos and clinician cohorts (e.g., first responders and emergency medicine physicians) to validate and extend our findings.

## 5 Conclusion

Neurologists rely on combinations of clinical signs, alongside subjective factors, when differentiating ES from FS. Collaborative diagnostic approaches and early referral to epileptologists or video EEG monitoring for cases with subtle or ambiguous semiology could reduce diagnostic delays and optimize management. The methodological framework and key findings presented here offer a valuable foundation for future research focused on validation, refinement, and the development of clinical decision‐support tools to enhance diagnostic accuracy in real‐world settings.

## Author Contributions

Subramanian Muthusamy: conceptualization, writing–original draft, methodology, writing–review and editing, data curation, formal analysis, visualization. Udaya Seneviratne: supervision, methodology, writing–review and editing, conceptualization. Henry Ma: writing–review and editing, supervision. Thanh G. Phan: supervision, conceptualization, writing–review and editing, formal analysis, methodology.

## Peer Review

The peer review history for this article is available at https://publons.com/publon/10.1002/brb3.70866

## Supporting information




**Supporting Table 1**: ‐ Self‐Assessed Rater Diagnostic Certainty by Case ID

**Supporting Table 2**: ‐ Self‐Assessed Rater Diagnostic Certainty by Seizure Classification

**Supporting Fig. 1**: – Clustering of clinical signs into two groups based on composite clinician rater response to question 2. Here the clustering is shown based on clinician's experience as a neurologist, The ‘basis heatmap’ represents the clustering of clinical signs into groups. The ‘coefficient heatmap’ represents the ‘weights’ attributed to each patient. The ‘consensus heatmap’ visualizes the stability and consistency of clustering of the results across multiple NMF runs.

**Supporting Fig. 2**: ‐ Top ten terms for topics 1 and 2 plotted against feature importance values. Note that feature importance values do not have units. Higher values indicate higher affinity towards a particular group.

**Supporting Fig. 3**: – Top ten terms for Topics 1 and 2 for different experience groups. Note that relative feature importance values do not have units of measurement. Higher values indicate higher affinity towards a particular group.

## Data Availability

The data that support the findings of this study are available on request from the corresponding author, once ethics approval is obtained.

## Appendix 1 Template of Survey Questions

### 1.1

**Review the accompanying video and answer the questions below**:

1. Describe the clinical event shown in the accompanying video
2. Select all of the clinic signs that are observed in this video:
3. Eye closure during event
4. Eyes open during event
5. Crying during event
6. Hyperventilation
7. Head version (tonic head turn to one side)
8. Side‐to‐side head shaking
9. Automatisms (oral or manual)
10. Tonic or dystonic posturing of limbs
11. Asynchronous limb jerking
12. Synchronous limb jerking
13. Pelvic thrusting
14. Fluctuating time course (waxing/waning)
15. Continuous time course
16. Select the diagnosis that best describes the accompanying video:

a. Epileptic seizure
b. Functional seizure (FS or pseudoseizure or psychogenic nonepileptic seizures)
c. Other
d. Unsure

1. How certain are you about your diagnosis?
2. Sliding scale from 0%–100%
