# Supplementary material for: How Neurologists Combine Clinical Signs and Subjective Factors to Diagnose Epileptic and Functional Seizures: Insights From Seizure Video Analysis
Source: Brain Behav. 2025 Sep 16;15(9):e70866. doi: 10.1002/brb3.70866 (PMC12441005; doi:10.1002/brb3.70866)
Supplement: Supplementary file 1 — Supporting Table 1: ‐ Self‐Assessed Rater Diagnostic Certainty by Case ID [file BRB3-15-e70866-s003.docx]

**Supplementary Table 1** - Self-Assessed Rater Diagnostic Certainty by Case ID

| **Case ID** | **Median %** | **Mean %** |
| --- | --- | --- |
| 2 | 98 | 92 |
| 15 | 97 | 93 |
| 22 | 96 | 91 |
| 5 | 90 | 84 |
| 21 | 88 | 80 |
| 14 | 84 | 83 |
| 19 | 84 | 79 |
| 12 | 81 | 80 |
| 1 | 81 | 81 |
| 6 | 81 | 81 |
| 10 | 80 | 74 |
| 16 | 80 | 65 |
| 8 | 79 | 76 |
| 9 | 79 | 70 |
| 3 | 76 | 78 |
| 13 | 76 | 78 |
| 17 | 76 | 66 |
| 11 | 71 | 61 |
| 18 | 70 | 60 |
| 4 | 68 | 59 |
| 20 | 66 | 52 |
| 7 | 64 | 62 |
