# Supplementary material for: How Neurologists Combine Clinical Signs and Subjective Factors to Diagnose Epileptic and Functional Seizures: Insights From Seizure Video Analysis
Source: Brain Behav. 2025 Sep 16;15(9):e70866. doi: 10.1002/brb3.70866 (PMC12441005; doi:10.1002/brb3.70866)
Supplement: Supplementary file 2 — Supporting Table 2: ‐ Self‐Assessed Rater Diagnostic Certainty by Seizure Classification [file BRB3-15-e70866-s001.docx]

**Supplementary Table 2** - Self-Assessed Rater Diagnostic Certainty by Seizure Classification

| **Seizure classification** | **Median %** | **Mean %** |
| --- | --- | --- |
| FBTCS | 97 | 92 |
| GTCS | 97 | 93 |
| Hypermotor FS | 86 | 83 |
| Focal motor tonic | 84 | 79 |
| FBTCS* | 83 | 79 |
| Mixed FS | 80 | 74 |
| Complex motor FS | 79 | 70 |
| Generalized motor tonic | 79 | 76 |
| Hypermotor ES | 71 | 61 |
| Focal motor automatisms | 68 | 56 |
| Focal motor* | 68 | 59 |

*Legend: FS: psychogenic nonepileptic seizures, FBTCS: focal to bilateral tonic clonic seizure; FBTCS*: focal to bilateral tonic clonic seizure, where only the initial 30s period (before secondary generalization is shown to the rater), Focal motor* (mixed phenomenology including initial hypermotor phenomena followed by manual automatisms)*
