# Supplementary material for: How Neurologists Combine Clinical Signs and Subjective Factors to Diagnose Epileptic and Functional Seizures: Insights From Seizure Video Analysis
Source: Brain Behav. 2025 Sep 16;15(9):e70866. doi: 10.1002/brb3.70866 (PMC12441005; doi:10.1002/brb3.70866)
Supplement: Supplementary file 3 — Supporting Fig. 1: – Clustering of clinical signs into two groups based on composite clinician rater response to question 2. Here the clustering is shown based on clinician's experience as a neurologist, The ‘basis heatmap’ represents the clustering of clinical signs into groups. The ‘coefficient heatmap’ represents the ‘weights’ attributed to each patient. The ‘consensus heatmap’ visualizes the stability and consistency of clustering of the results across multiple NMF runs. [file BRB3-15-e70866-s002.docx]

**Supplementary Figure 1** – Clustering of clinical signs into two groups based on composite clinician rater response to question 2. Here the clustering is shown based on clinician’s experience as a neurologist, The ‘basis heatmap’ represents the clustering of clinical signs into groups. The ‘coefficient heatmap’ represents the ‘weights’ attributed to each patient. The ‘consensus heatmap’ visualizes the stability and consistency of clustering of the results across multiple NMF runs.

| Group 1: 0-5 years’ experience | Group 2: 5-10 years’ experience | Group 3: >10 years’ experience |
| --- | --- | --- |
| 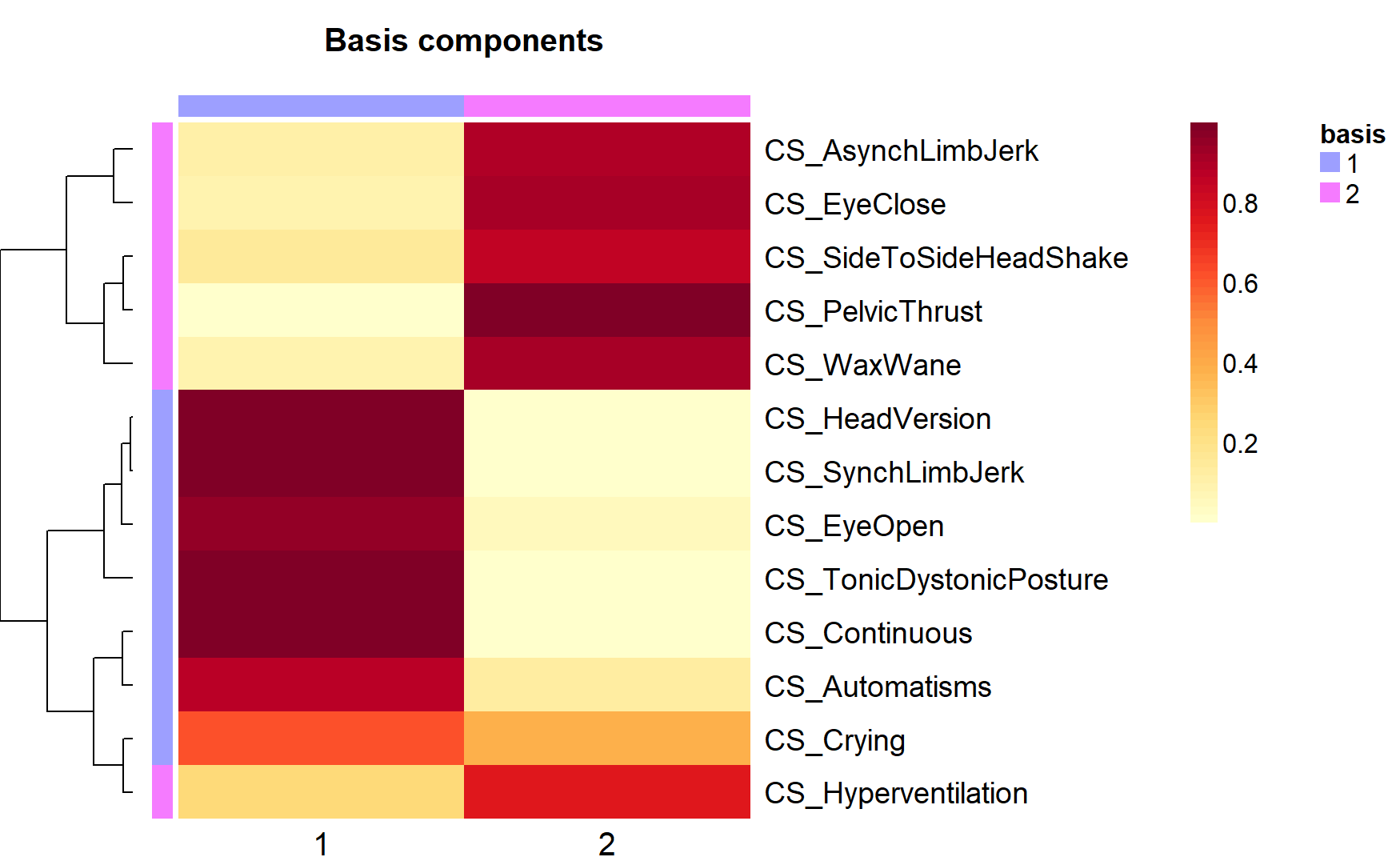 | 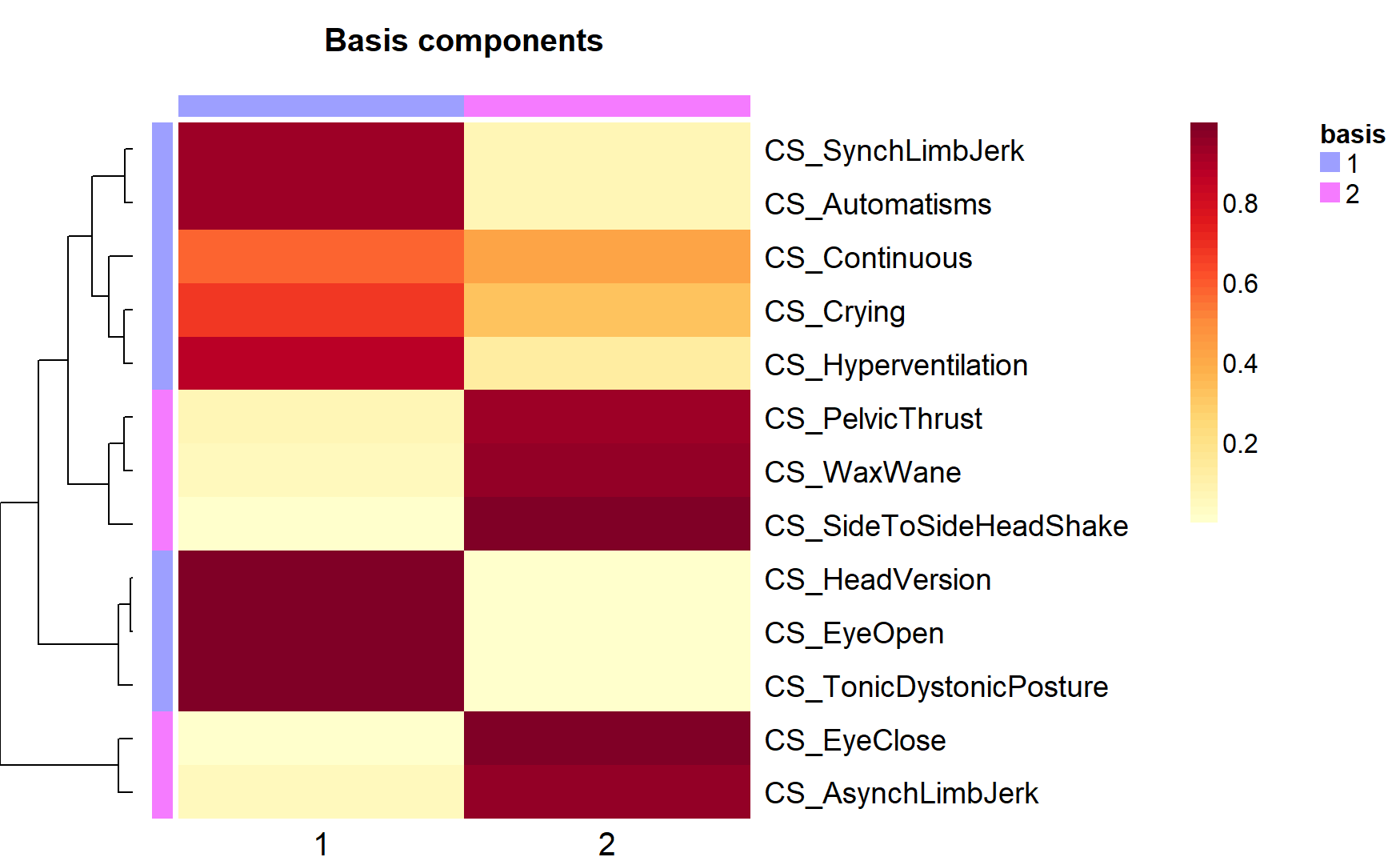 | 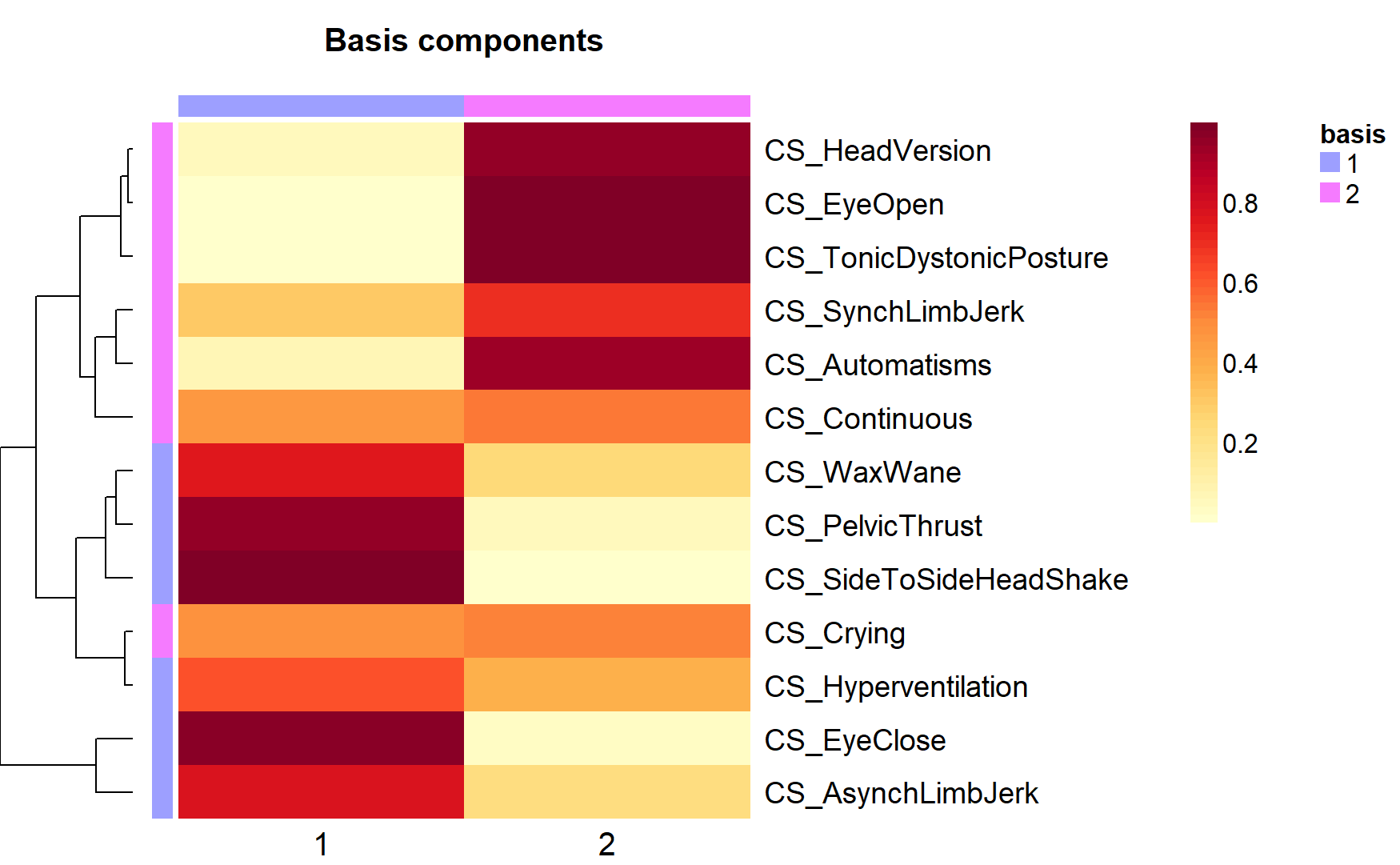 |
| 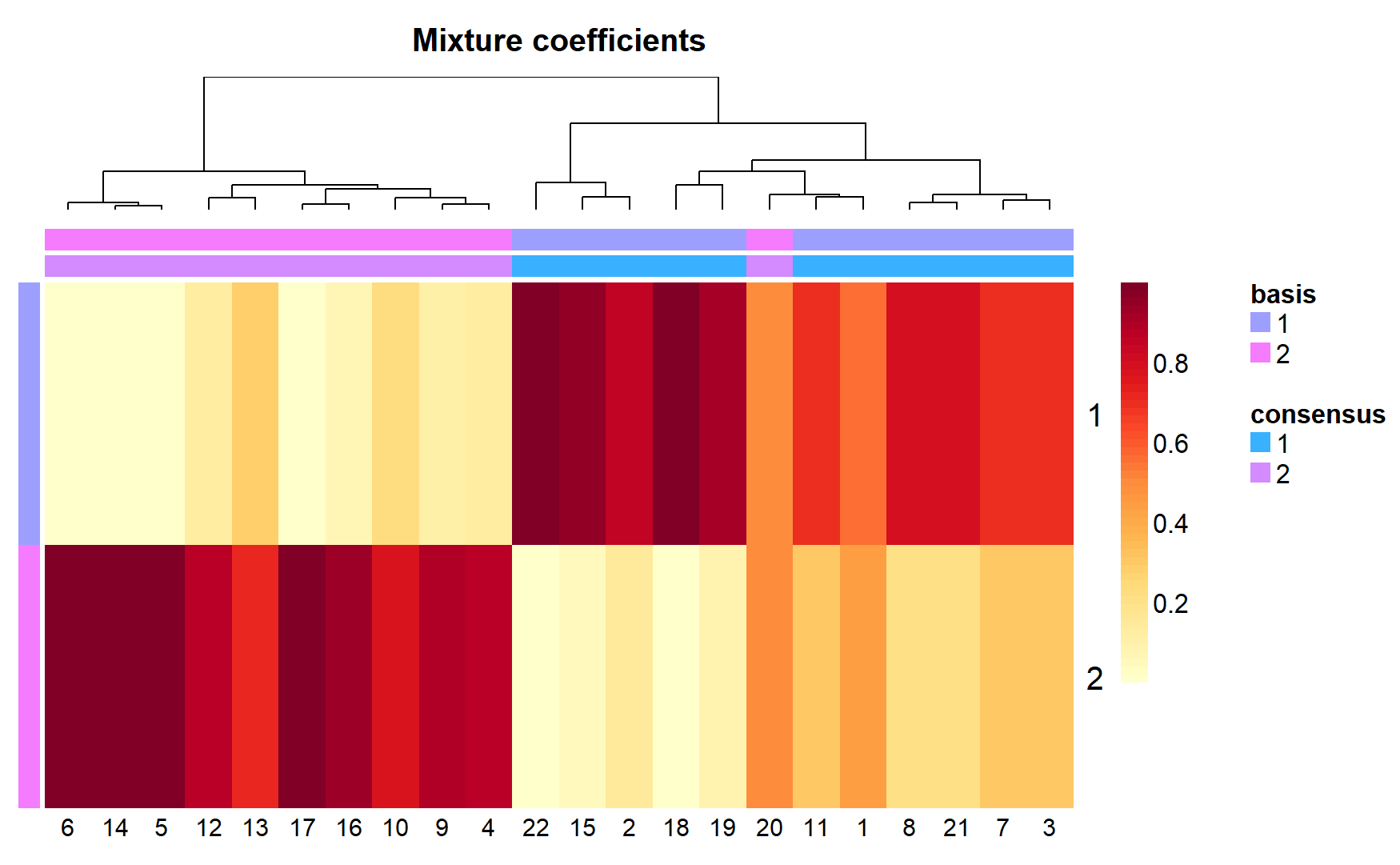 | 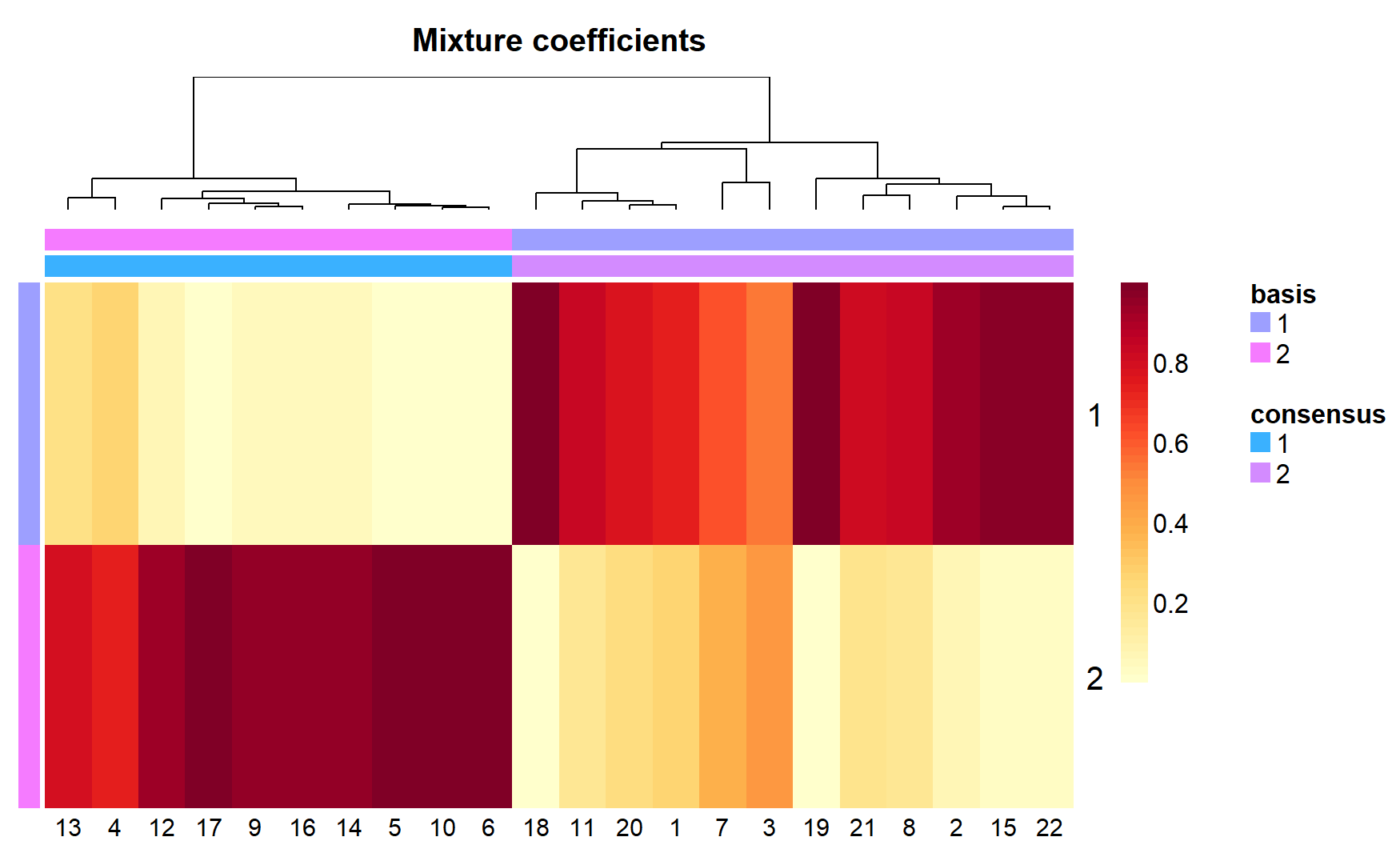 | 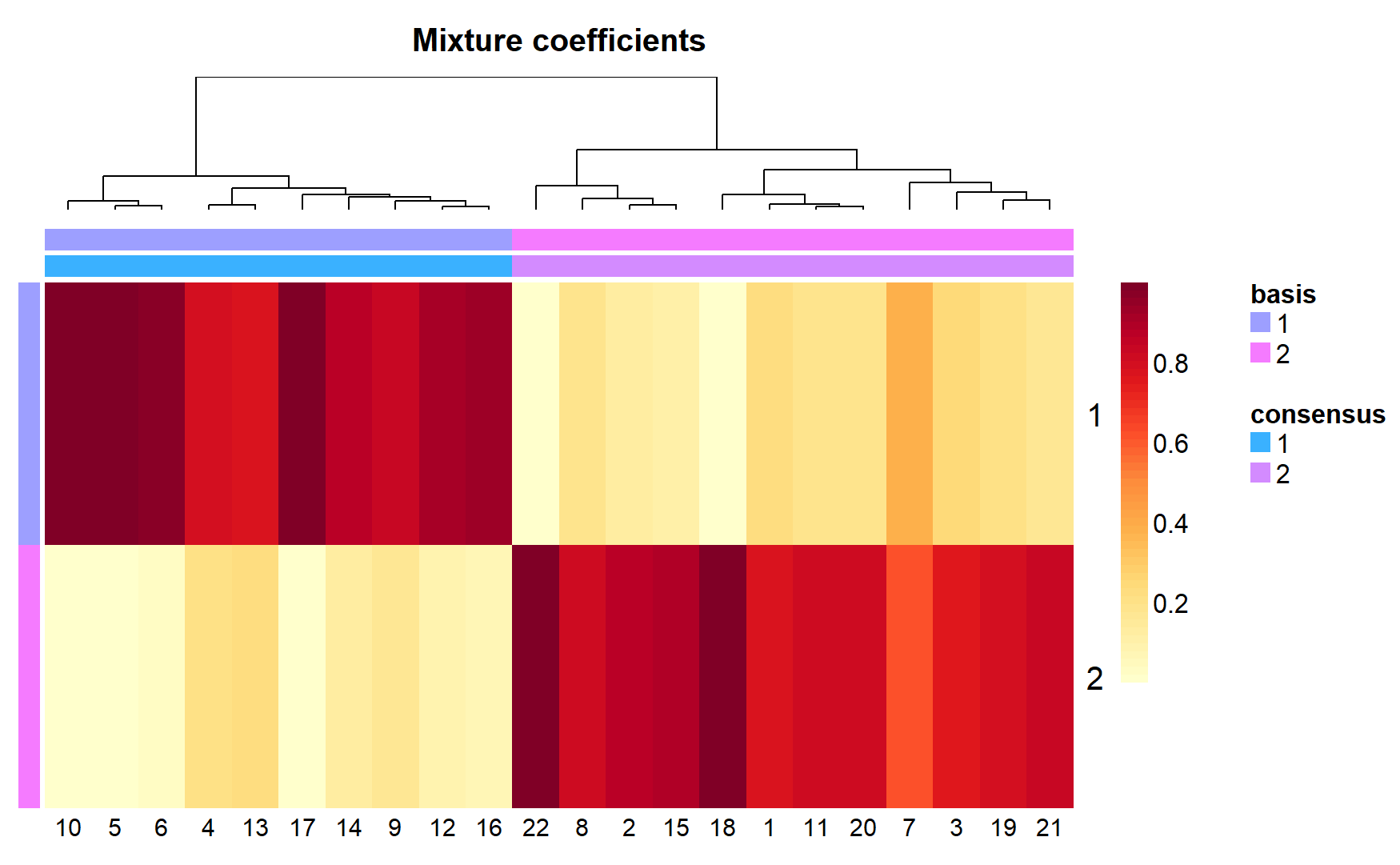 |
| 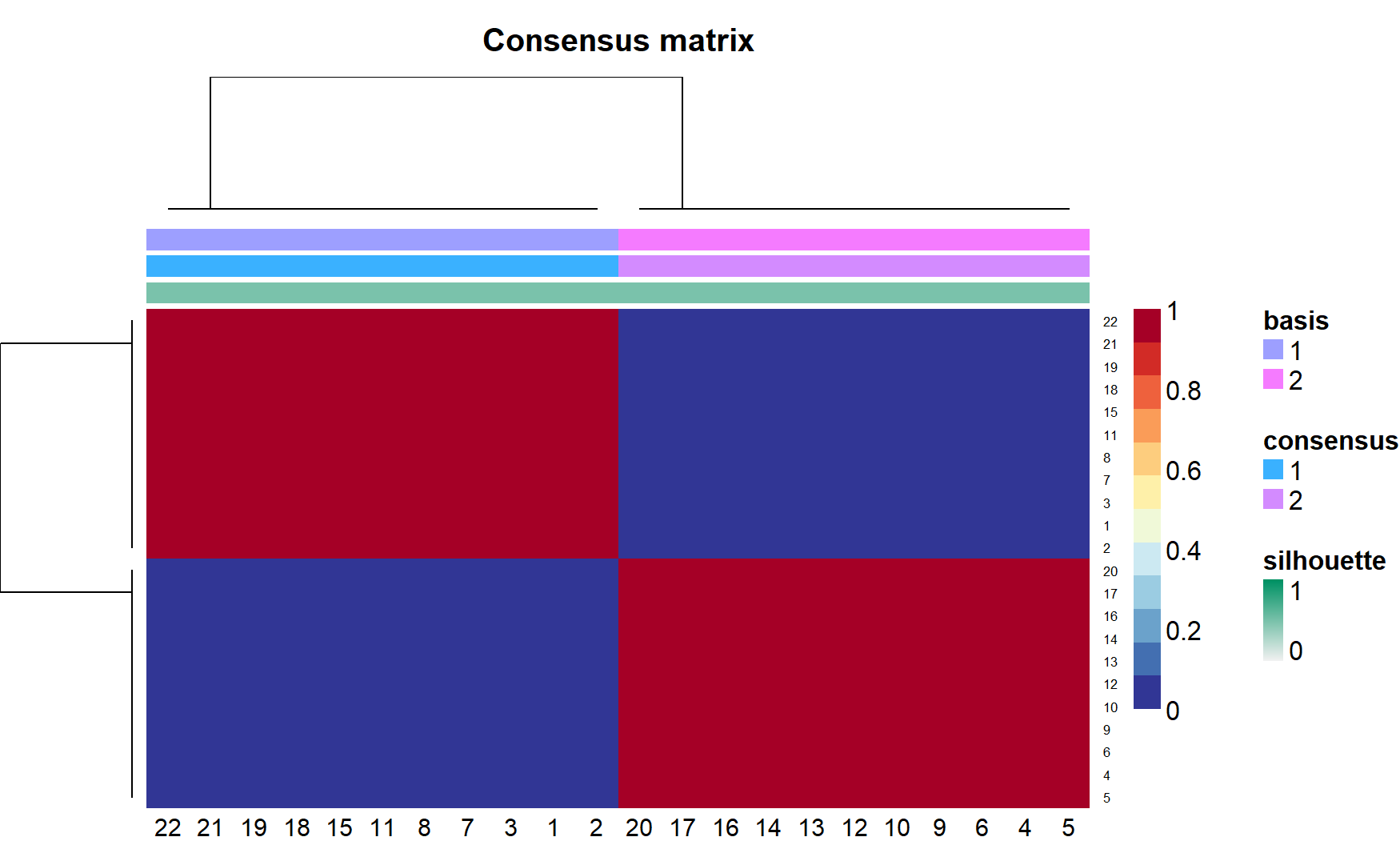 | 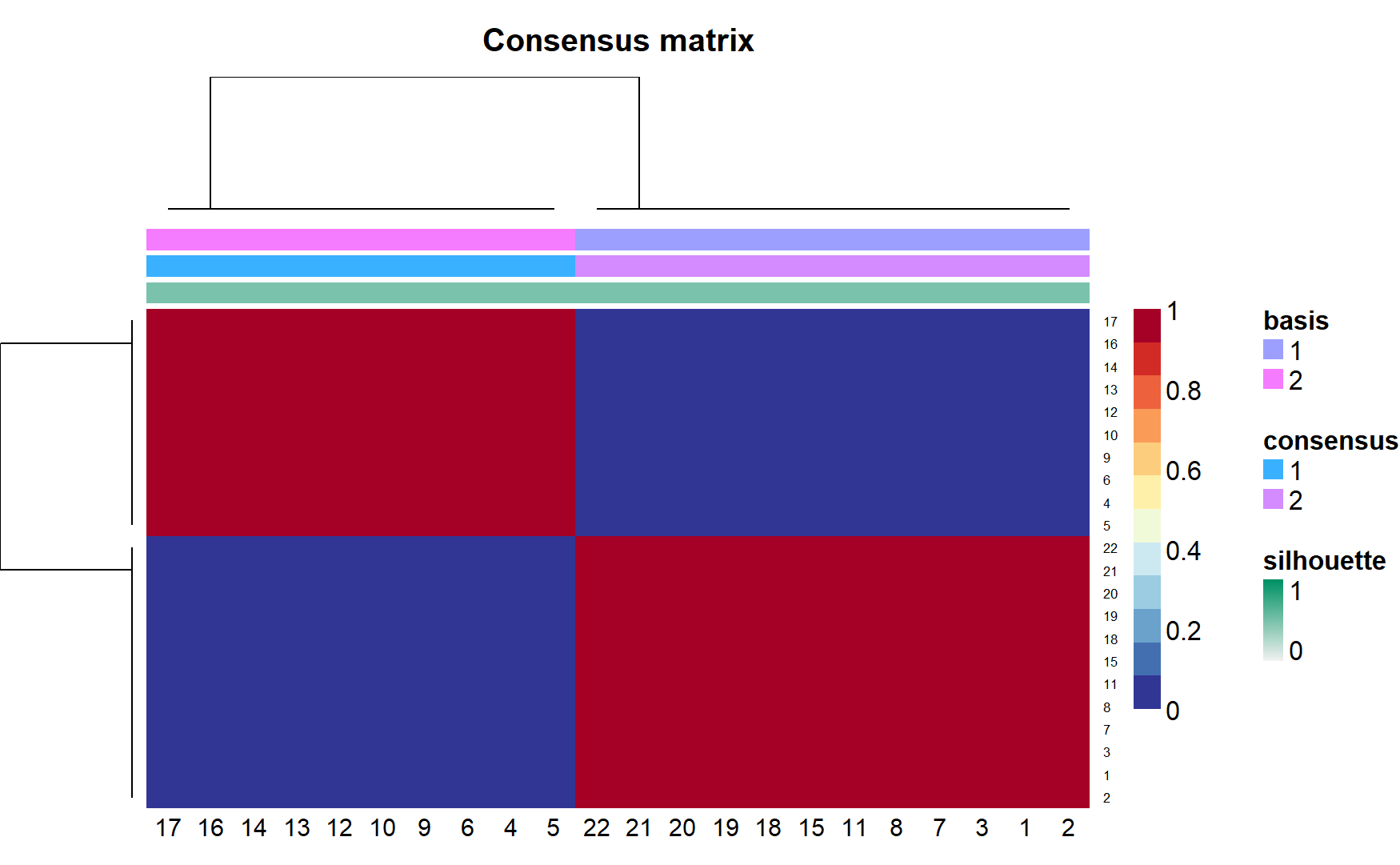 | 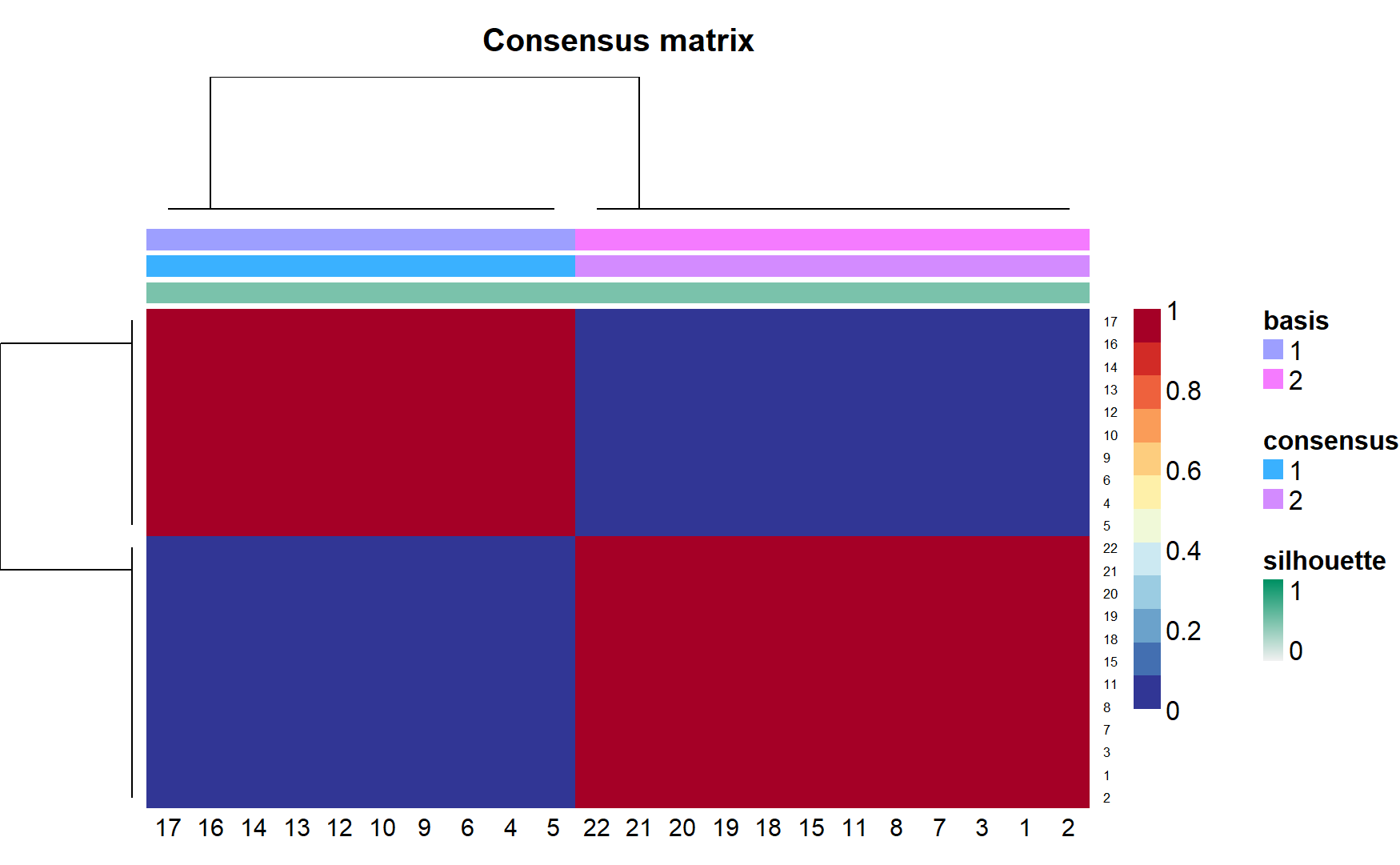 |

**Supplementary Figure 2** - Top ten terms for topics 1 and 2 plotted against feature importance values. Note that feature importance values do not have units. Higher values indicate higher affinity towards a particular group.


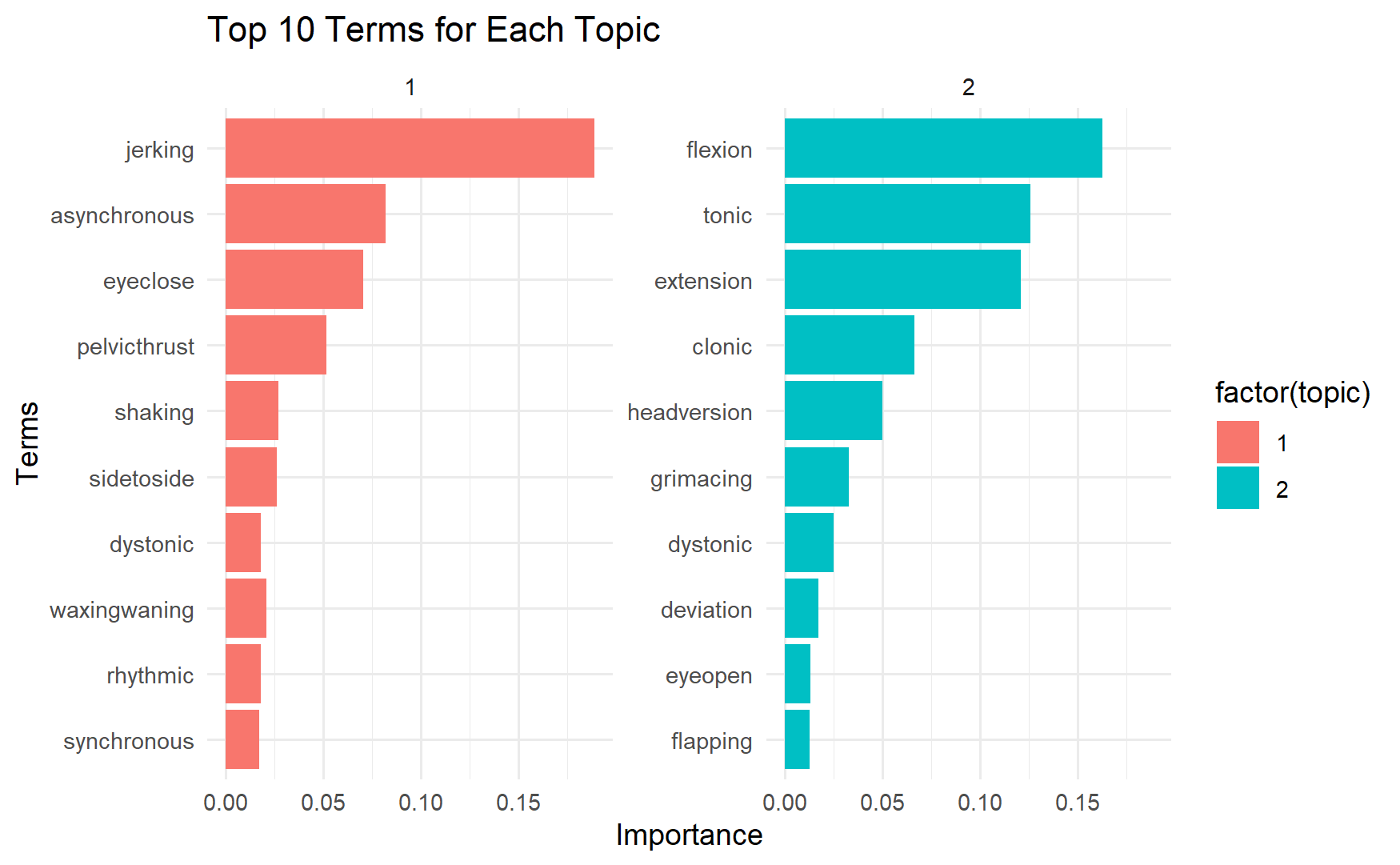


**Supplementary Figure 3** – Top ten terms for Topics 1 and 2 for different experience groups. Note that relative feature importance values do not have units of measurement. Higher values indicate higher affinity towards a particular group.

| 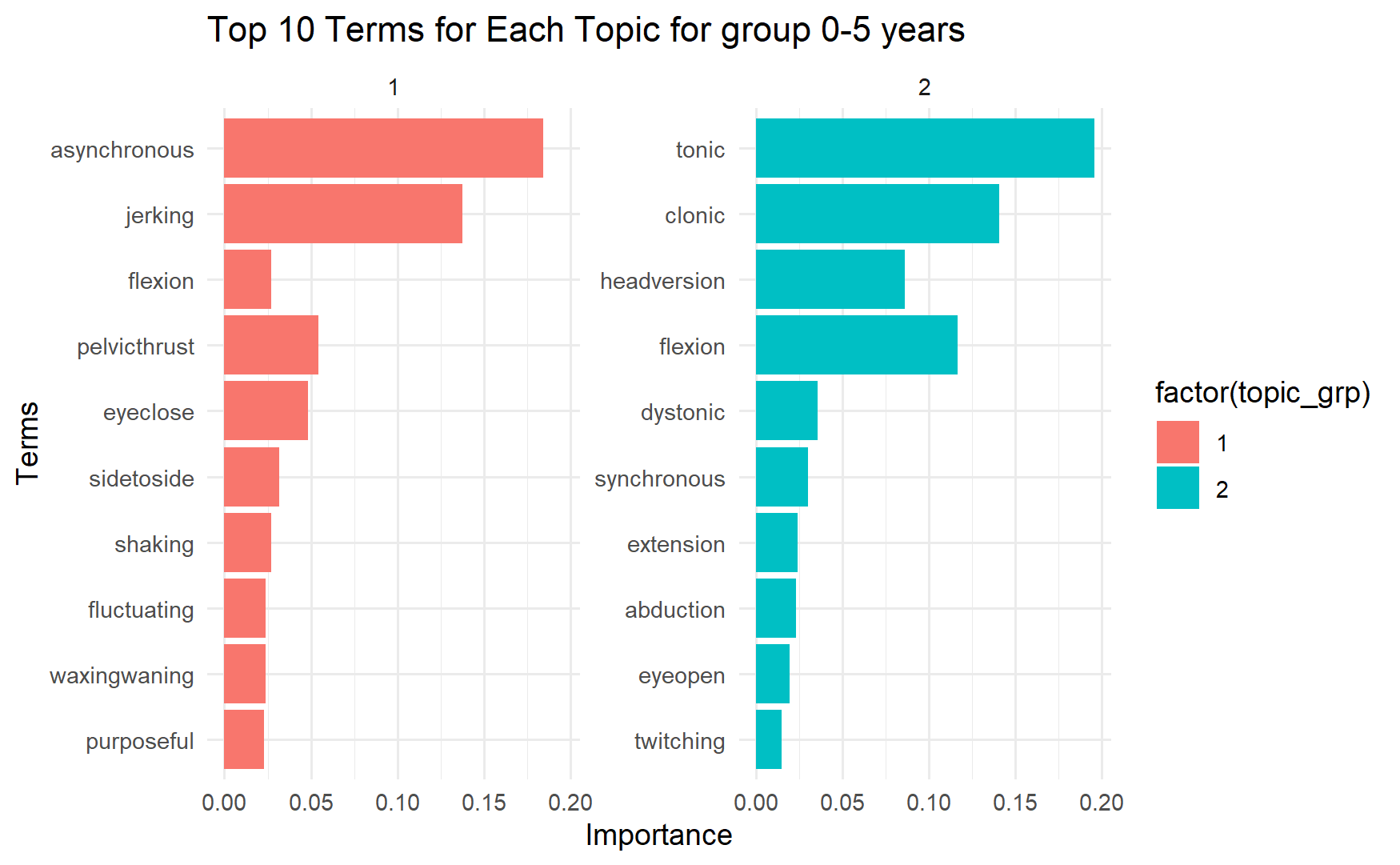 Group 1: 0-5 years of experience |
| --- |
| 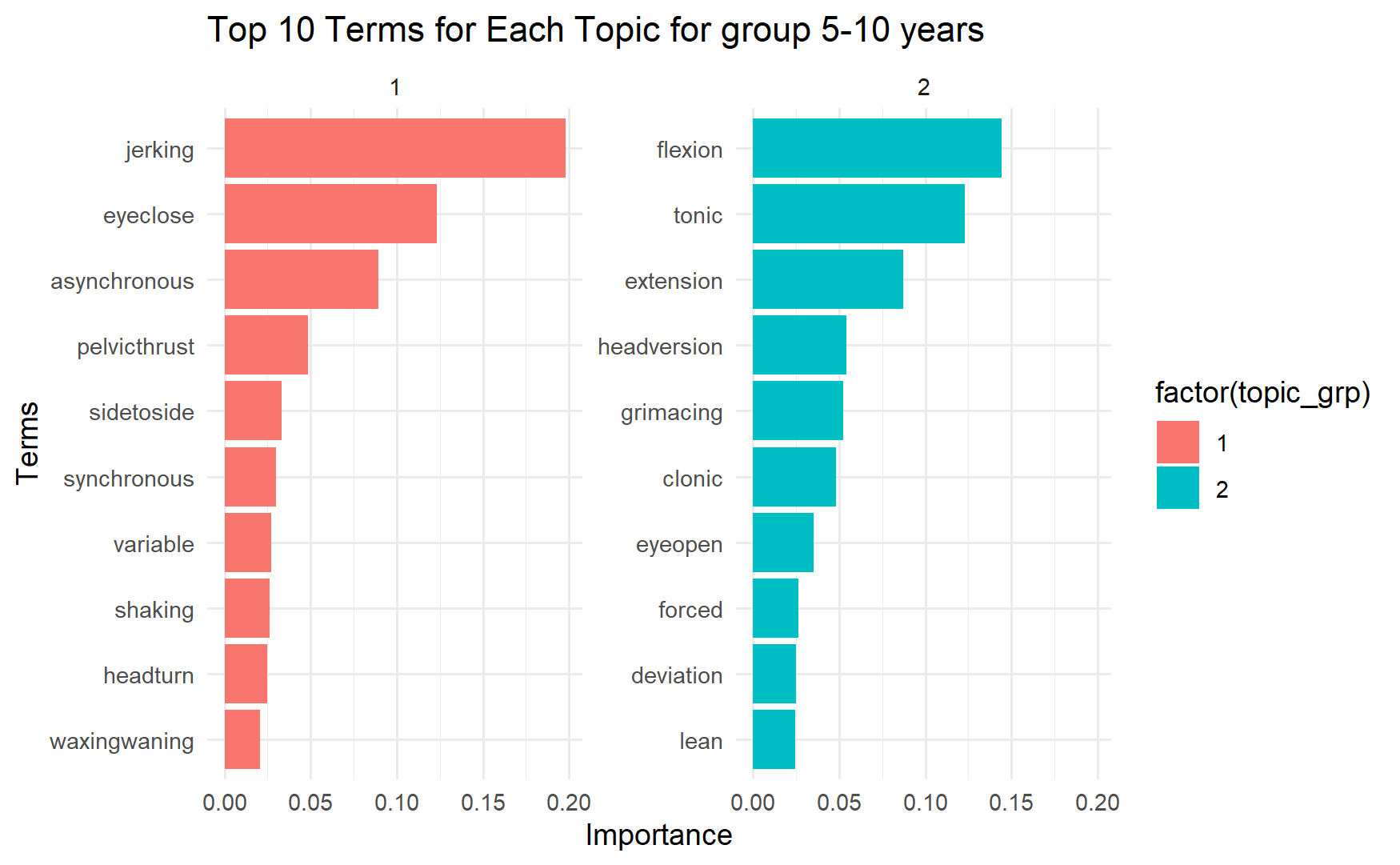Group 2: 5-10 years of experience. |
| 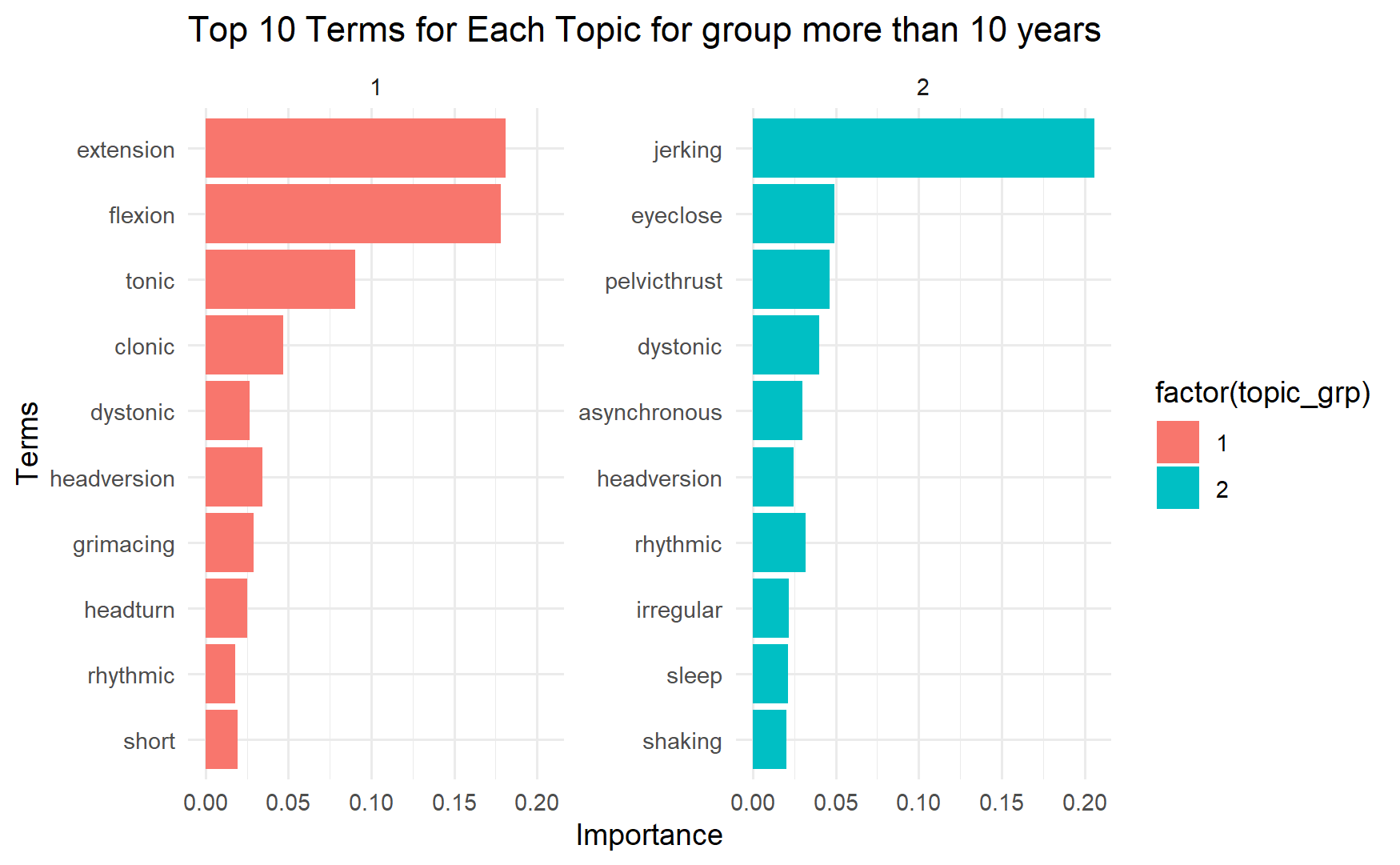Group 3: More than 10 years of experience |
